# Exogenous lipase administration alters gut microbiota composition and ameliorates Alzheimer’s disease-like pathology in APP/PS1 mice

**DOI:** 10.1038/s41598-022-08840-7

**Published:** 2022-03-21

**Authors:** Ariane Menden, Davane Hall, Coral Hahn-Townsend, Courtney A. Broedlow, Utsav Joshi, Andrew Pearson, Fiona Crawford, James E. Evans, Nichole Klatt, Stefan Crynen, Michael Mullan, Ghania Ait-Ghezala

**Affiliations:** 1 Roskamp Institute, 2040 Whitfield Avenue, Sarasota, FL 34243 USA; 2 Open University, Walton Hall, Kents Hill, Milton-Keynes, MK7 6AA UK; 3 Division of Surgical Outcomes and Precision Medicine Research, Department of Surgery, University of Minnesota, 420 Delaware Street SE, Minneapolis, MN 55455 USA; 4 James A. Haley Veterans’ Hospital, 13000 Bruce B. Downs Boulevard, Tampa, FL 33612 USA

**Keywords:** Alzheimer's disease, Biomarkers, Microbiome, Diagnostic markers, Molecular medicine, Translational research

## Abstract

Alzheimer’s disease (AD) represents the most common form of dementia in the elderly with no available disease modifying treatments. Altered gut microbial composition has been widely acknowledged as a common feature of AD, which potentially contributes to progression or onset of AD. To assess the hypothesis that Candida rugosa lipase (CRL), which has been shown to enhance gut microbiome and metabolite composition, can rebalance the gut microbiome composition and reduce AD pathology, the treatment effects in APPswe/PS1de9 (APP/PS1) mice were investigated. The analysis revealed an increased abundance of *Acetatifactor* and *Clostridiales vadin* *BB60 *genera in the gut; increased lipid hydrolysis in the gut lumen, normalization of peripheral unsaturated fatty acids, and reduction of neuroinflammation and memory deficits post treatment. Finally, we demonstrated that the evoked benefits on memory could be transferred via fecal matter transplant (FMT) into antibiotic-induced microbiome-depleted (AIMD) wildtype mice, ameliorating their memory deficits. The findings herein contributed to improve our understanding of the role of the gut microbiome in AD’s complex networks and suggested that targeted modification of the gut could contribute to amelioration of AD neuropathology.

## Introduction

Dementia affects more than 50 million people worldwide with AD being the most common form. Despite extensive research on the neuropathological hallmarks of AD, no disease-modifying treatments have been developed which can stop or slowing progression of the disease. Thus far, almost all clinical trials attempting to treat AD by removing amyloid plaques or inhibiting γ-secretase activity have failed, which has led to investigations of other treatment strategies^1^.

One such novel target is the gut microbiome, due to its ability to systemically impact host physiology and metabolism^2–4^. It has been demonstrated that gut microbes play an important role in brain-related disorders such as Parkinson’s disease and multiple sclerosis affecting disease onset and severity of neuropathology^5^. In AD patients, gut dysbiosis has been identified and denoted by a decrease in the bacterial phyla *Firmicutes* and *Actinobacteria* and increased abundance of *Bacteroidetes*^6–8^. Similarly, mouse models of AD-like pathology have shown significant differences in the microbial composition when compared to wild-type controls^9,10^. Furthermore, decreased pathology has been observed in germ-free APP/PS1 mice when compared to conventionally raised controls emphasizing the potential contribution of the gut microbiome to AD pathology^10^. Therefore, these and other studies provide evidence to support the hypothesis that the gut contributes to AD pathology via the gut-brain axes and that compositional enhancement may reduce AD pathology^11^.

Administration of exogenous enzymes such as proteases, amylases, and lipases have been shown to alter the gut microbiome composition and to improve digestive performance of healthy individuals and patients with, for instance, cystic fibrosis or pancreatic insufficiency^12–14^. In a recent study we demonstrated that orally administered CRL altered gut microbial β-diversity and promoted growth of bacterial species in Wt mice such as *A.muciniphila* and *Anaerostipes*, which have been associated with anti-inflammatory and anti-diabetic effects^15–20^. CRL has a broad specificity range for triglyceride and cholesterol ester hydrolysis, which result in the release of fatty acids, cholesterol, and glycerol into the gut lumen^13,21^. The lipase hydrolysis products can be absorbed by the host or gut microbiota to enhance their growth and metabolite production^22–25^. The microbial associated metabolites can exert anti-inflammatory and anti-bacterial properties potentially ameliorating AD-like pathology^22–25^. Furthermore, breakdown products directly absorbed by the host can impact lipid and cholesterol homeostasis, which have been associated with AD pathology and represent pivotal targets to potentially alter AD pathology^26^.

Therefore, it was hypothesized that CRL administration can improve gut microbiome and metabolome composition, which might lead to amelioration of AD pathology and aberrant behavior. In *Study 1*, the effects of CRL treatment on the gut, periphery, and brain in transgenic APP/PS1 mice, a mouse model of AD, was examined and compared to C57BL/6J (Wt) mice. In addition, we used the fecal matter of those mice to assess whether the CRL treatment-dependent enhancement of memory was transferable into AIMD Wt mice to verify the gut as the origin of the observed beneficial effects in *Study 2*.

## Results

To investigate the underlying hypothesis that the CRL treatment-dependent gut microbial and metabolic changes may ameliorate AD-like pathology, the investigation (*Study 1*) was subdivided into three analysis sections: gut, peripheral and brain-related changes.

### CRL treatments alter gut microbiome and metabolite composition

**Figure 1:**
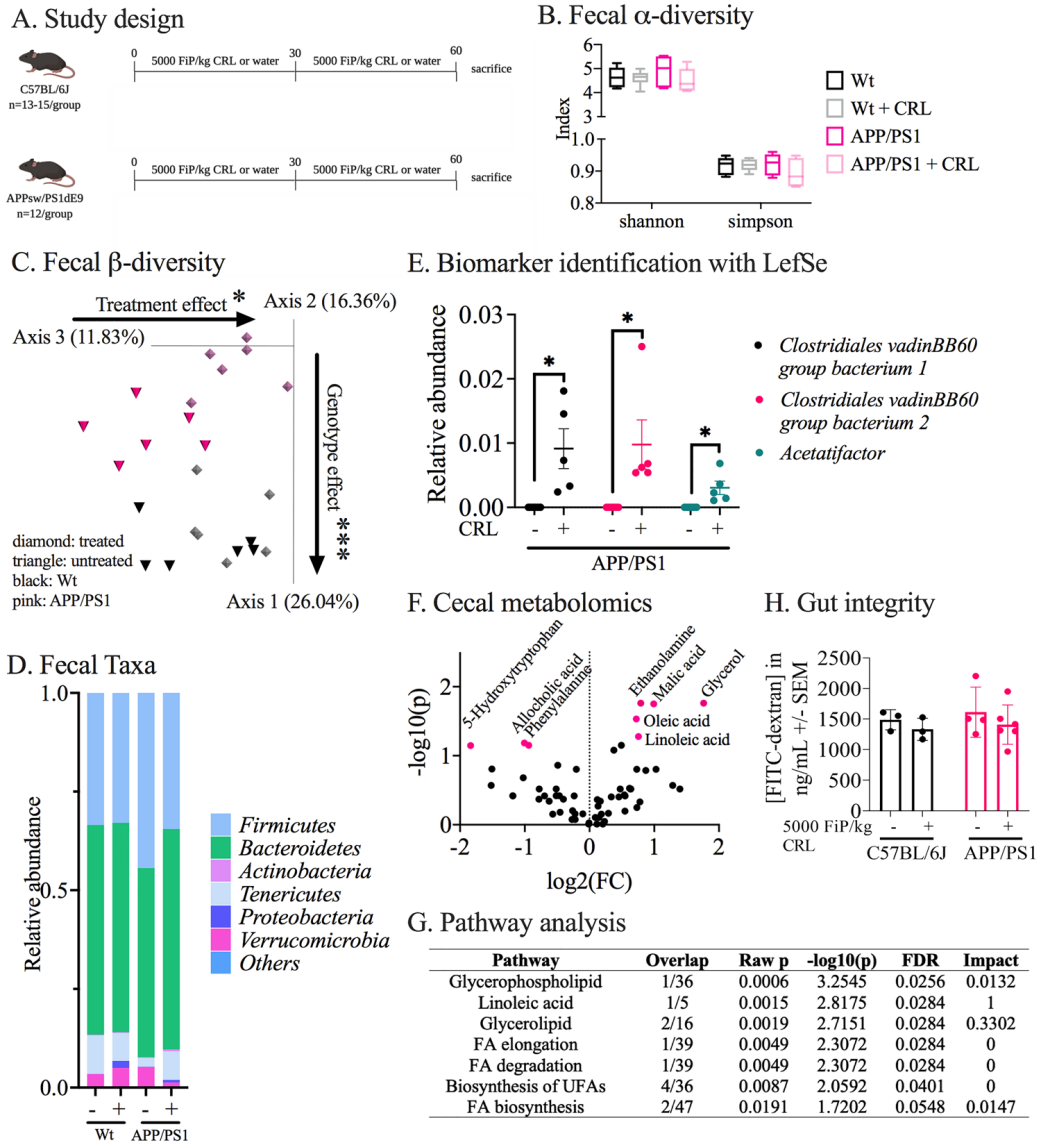
Gut-related changes through CRL treatment in APP/PS1 and Wt mice. **A** Study design: Wt (n = 13–15, 8 months of age) and APP/PS1 (n = 12, 8 months of age) received 5000 FIP/kg CRL for two consecutive months or regular water before sacrifice. **B** α-diversity analysis in fecal matter analyzed by Shannon and Simpson index. **C**. β-diversity analysis by Bray–Curtis dissimilarity and distances of fecal matter. **D** Taxonomy analysis of phyla abundance in fecal matter by alignment with Greengenes database. **E** Biomarker identification via LEfSe on genus level identified significant genera driving microbial changes in APP/PS1 treated versus untreated animals. **F** Metabolomics analysis of cecal matter of APP/PS1 groups showed increased abundance of metabolites that are associated with lipid digestion. **G** Pathway analysis of identified metabolites to determine key pathways activated through lipase administration revealing pathways associated with lipid degradation. H. Gut integrity analysis by transition measurement of oral administered FITC-dextran dye into plasma did not show a significant difference between treatment groups. Significance for α-diversity was assessed by Kruskal–Wallis H test and pairwise comparisons, for β-diversity with PERMANOVA. Cecal metabolomic data and LEfSe results were analyzed using 2-way ANOVA and FDR correction. Gut integrity was evaluated using 2-way ANOVA. Significance: *p < 0.05, **p < 0.01.

In *Study 1,* we examined the hypothesis that the most prominent changes in response to CRL treatment would be observed in the gut environment where the enzyme was physically present and active (Fig. 1A). Accordingly, the fecal microbiome, the gut metabolome, as well as gut integrity in the gut post treatment were investigated and compared to untreated age-matched controls. In the fecal microbiome, no changes in α-diversity were observed between groups after 2 months of treatment, which was consistent across the cecal (2 months of treatment, Supplementary Fig. 1A) and fecal data (0, 1 and 2 months of treatment; Fig. 1B, Supplementary Fig. 1B) between Wt and APP/PS1 (genotype-dependent) as well as treated and untreated animals (treatment-dependent). By contrast, β-diversity showed genotype-dependent and treatment-dependent dissimilarity in both fecal (p = 0.001 and p = 0.031, respectively, Fig. 1C) and cecal matter (p = 0.001 and p = 0.017, respectively, Supplementary Fig. 1A) post 2 months of treatment. When investigating longitudinal changes of fecal β-diversity (0, 1 and 2 months of treatment), dissimilarities were observed between all groups (Supplementary Fig. 1A1B), changes were most pronounced between genotypes (p = 0.001, Supplementary Fig. 1B) and treatment groups (APP/PS1: p = 0.001; Wt: p = 0.017, Supplementary Fig. 1B). Taxonomic summaries of fecal samples on the phyla level, after 2 months of treatment, indicated differences between untreated Wt and APP/PS1 animals while the fecal microbiome of treated APP/PS1 was reshaped towards Wt taxa composition (Fig. 1D). In addition, two months of CRL treatment increased *Proteobacteria* levels in both genotypes but appeared to be more pronounced in treated Wt mice in fecal samples (Supplementary Fig. 5D; Fig. 1D). Finally, Linear discriminant analysis Effect Size (LEfSe) was applied for analysis of microbes in fecal samples of APP/PS1 mice after 2 months of treatment, which identified *Clostridiales vadin BB60 group* and *Acetatifactor* genera to be significantly increased in treated animals (Fig. 1E). Next, gas-chromatography mass-spectrometry (GCMS) was used to examine whether the fecal microbial shifts were associated with alteration in the cecal gut metabolome after two months of treatment. The identified increase of the *Acetatifactor* genus in treated APP/PS1 mice (which has been discovered in an obese mouse^27^) was hypothesized to have occurred in response to lipase-dependent fatty acid release and other potential alterations of the gut metabolome (Fig. 1F). Therefore, treatment-dependent changes of the gut metabolite composition were investigated in APP/PS1 mice. Three metabolites were significantly decreased (5-hydroxytryptophan: fold change (FC) = 0.280, p_false discovery rate (FDR)_ = 0.071; allocholic acid FC = 0.498, p_FDR_ = 0.065; and phenylalanine FC = 0.522, p_FDR_ = 0.071), while five metabolites were significantly increased (malic acid, FC = 1.988, p_FDR_ = 0.018; ethanolamine, FC = 1.732, p_FDR_ = 0.017; linoleic acid, FC = 1.690, p_FDR_ = 0.052; oleic acid, FC = 1.650, p_FDR_ = 0.029; and glycerol, FC = 3.393, p_FDR_ = 0.017; Fig. 1F). The associated metabolome pathway analysis further identified pathways associated with unsaturated fatty acids and lipid metabolism supporting the hypothesis that CRL administration elevates fatty acid release in the gut lumen of APP/PS1 mice (Fig. 1G).

Finally, gut barrier integrity was assessed (Fig. 1H). No differences between treated and untreated animals or APP/PS1 and Wt animals were observed. Therefore, the gut analysis supported the hypothesis that CRL treatment induces changes in the gut lumen that may be beneficial to the host.

### CRL treatment normalizes peripheral levels of unsaturated fatty acids

**Figure 2:**
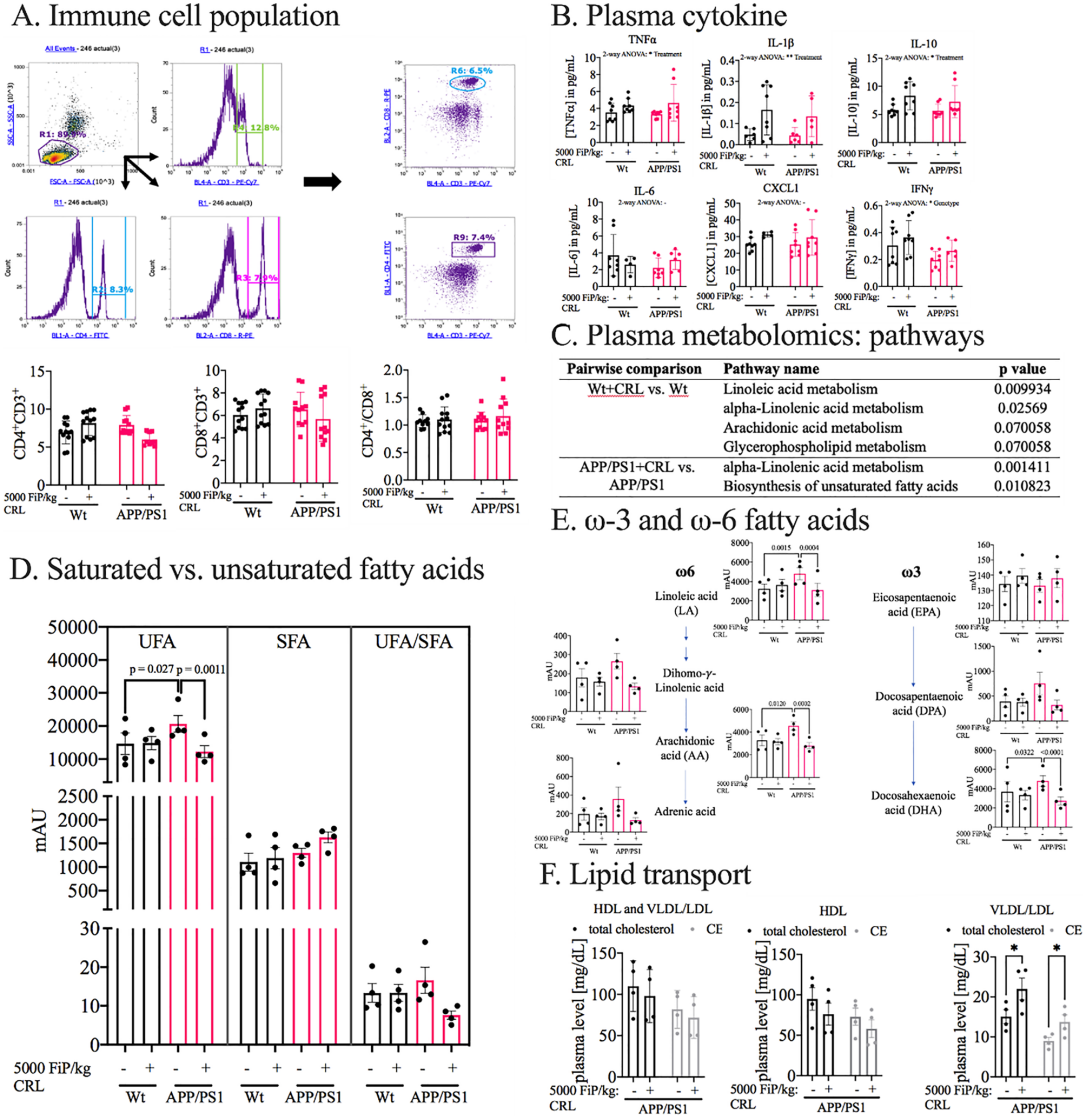
Peripheral impact of CRL treatment. **A** Quantification of immune cell population by flow cytometry analysis. No difference was observed in CD4+CD3+, CD8+CD3+ and their ratio, respectively. **B** Peripheral inflammation measured by plasma cytokine and chemokine levels. No biologically-relevant significant changes could be determined. **C** Pathway analysis of untargeted plasma metabolomics data indicating significant changes in UFA and glycerophospholipid metabolism. **D** Investigation of subclasses of UFA, SFA and UFA/SFA revealed an UFA specific elevation in APP/PS1 mice and reduction post treatment. **E** ω-3 and ω-6 fatty acid levels measured in plasma. All subtypes showed significant or trending normalization of UFA level post CRL treatment in APP/PS1 mice. **F** HDL and VLDL/LDL cholesterol and cholesterol esters to analyze CRL’s effect on lipid transport. VLDL/LDL cholesterol and cholesterol ester fraction were both significantly increased. Significance was assessed by 2-way ANOVA and post-hoc Tukey correction. Significance: *p < 0.05, **p < 0.01, ***p < 0.001.

Subsequently, it was investigated whether the identified gut alterations were reflected in the host’s peripheral circulation, which has been shown to be in direct exchange with the gut, through the gut-brain axes^28^. First, immune cell populations (T-helper: CD3+CD4+ and cytotoxic T cells: CD3+CD8+) were examined by flow cytometry (Fig. 2A). While no difference was measured between untreated Wt and APP/PS1 mice, a trend for decreased CD3+CD4+ and CD3+CD8+ populations was observed in treated APP/PS1 mice when compared to untreated animals. But, since the ratio was unaffected by treatment, compared to control groups the analysis suggested no treatment-dependent effects (Fig. 2A). Measurement of plasma cytokine levels assessed treatment-dependent activity changes of immune cells (Fig. 2B; Supplementary Fig. 2B). Although multiple cytokines showed treatment-dependent trends, the results had negligible biological relevance due to the overall low concentrations. Hence, the immunological axis was excluded from further investigations. Next, the metabolic axis was analyzed to determine the treatment-dependent changes in plasma by untargeted metabolomics. The pathway analysis revealed, for both genotypes, significant treatment-dependent alterations in pathways associated with unsaturated fatty acid metabolism (Fig. 2C). To identify further the direction of the identified alterations, the data was sub-grouped in total saturated (SFA) and unsaturated fatty acids (UFA, Fig. 2D). While no significant genotype-dependent and treatment-dependent effects in SFAs and UFAs-to-SFAs ratio were observed, UFA levels were significantly increased in untreated compared to treated APP/PS1 mice (p = 0.001, Fig. 2D). Further subdivision of UFAs in ω-3 and ω-6 fatty acids displayed the same trend in both sub-groups (ω-3: p = 0.0008; ω-6: p = 0.021), but the effect was more pronounced in ω-3 fatty acids (Supplementary Fig. 2A). These, results warranted the subsequent analysis of specific unsaturated fatty acid levels and their downstream products, which showed significant reduction of the associated metabolites in treated APP/PS1 mice in comparison to untreated APP/PS1 levels including linoleic (FC_APP/PS1_ = 0.651; p = 0.0002), arachidonic (FC_APP/PS1_ = 0.618; p = 0.0001) and docosahexaenoic acid (FC_APP/PS1_ = 0.569; p < 0.0001; Fig. 2E). Finally, it was investigated whether alterations in lipid transport favored the peripheral differences of UFA in APP/PS1 mice measured by high-density lipoproteins (HDL) and very low-density and low-density lipoprotein (VLDL/LDL) cholesterol plasma levels (Fig. 2F). Peripheral total cholesterol and cholesterol ester levels were unchanged, while cholesterol and cholesterol esters in HDL and VLDL/LDL fractions showed treatment-dependent divergent differences (Fig. 2F). Only VLDL/LDL cholesterol and cholesterol ester levels were significantly increased post treatment (cholesterol: FC = 1.459, p = 0.031; cholesterol ester: FC = 1.529, p = 0.031; Fig. 2F).

In summary, we showed that CRL treatment altered the microbial β-diversity in fecal and cecal specimen and elevated two *Clostridiales vadin BB60* genera as well as *Acetatifactor* abundance. Furthermore, we showed that cecal UFA levels of treated APP/PS1 mice were elevated, while peripheral UFA levels were normalized, and VLDL/LDL cholesterol and cholesterol esters were increased.

### CRL treatment affects glial cell marker levels and memory

**Figure 3:**
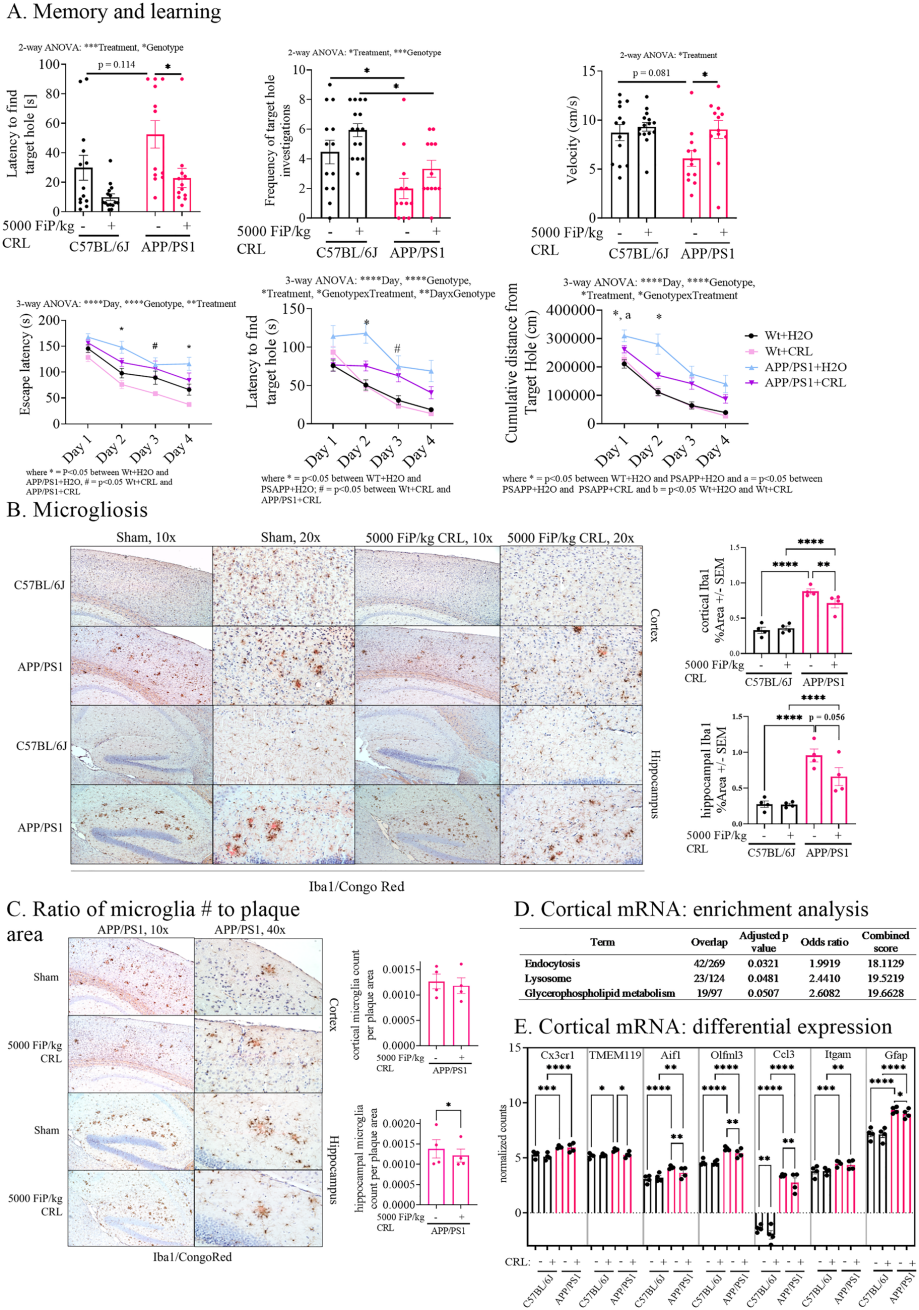
Brain pathological impact of CRL treatment. **A** Probe and acquisition trial parameters of Barnes maze to assess memory and learning. The probe trial showed a significant improvement for latency to find target hole as well as frequency of target hole investigations for treated mice and a trend for treated WT mice. The acquisition data suggested trends for improved learning in treated APP/PS1 mice. **B** Immunohistochemical analysis of treatment-dependent effects on microglia activation. Iba1/Congo Red stained cortex showed reduction in astrocytosis and microgliosis (× 20 magnification: four animals per group, four slides per animal, three images per tissue area). **C** Assessment of amyloid plaque size to associated microglia counts to determine changes in inflammatory response and plaque burden (× 40 magnification: four animals per group, four slides per animal, six images per tissue area). **D** Enrichment analysis of significant uncorrected cortical transcriptomic data of treated and untreated APP/PS1 mice suggesting microglia and astrocyte activity being altered as well as glycerophospholipid metabolism. **E** Transcriptomics analysis of brain cell markers of whole cortical tissue revealing treatment-dependent changes in microglia-specific genes in APP/PS1 mice. Significance of probe trial was determined by 1- or 2-way ANOVA and post-hoc Tukey analysis, dependent on the respective parameter, while acquisition data was analyzed using 3-way ANOVA and post-hoc Tukey analysis. 2-way ANOVA and Tukey correction were further applied for immunohistochemical results and 2-way ANOVA and FDR correction for transcriptomics analysis. Significance: *p < 0.05, **p < 0.01, ***p < 0.001.

Next, CRL’s treatment effect on memory and learning (Barnes maze), neuroinflammation (Glial Fibrillary Acidic Protein antibody (GFAP)/ ionized calcium-binding adapter molecule 1 (Iba1)/Congo Red staining), and gene expression (RNAseq) was examined. The Barnes maze acquisition trials mostly showed significant differences between untreated Wt and APP/PS1 animals and trends for treatment-dependent improvements (Fig. 3A; Supplementary Fig. 3B). The probe trial showed significant genotype-dependent differences for frequency to find the target hole and a trend for latency to find the target hole (latency: p = 0.114; frequency: p = 0.043; Fig. 3A; Supplementary Fig. 3A). Significant improvement of latency to find the target hole and a trend for improvement in frequency of target hole investigations was determined in treated APP/PS1 mice when compared to untreated littermates (latency: p = 0.025; frequency: p = 0.478; Fig. 3A; Supplementary Fig. 3A). Furthermore, CRL treated Wt mice showed a trend for improvement when compared to untreated animals (latency: p = 0.158; frequency: p = 0.325; Fig. 3A; Supplementary Fig. 3A/B). In addition, untreated APP/PS1 mice exhibited decreased velocity results in both acquisition and probe days, which was normalized upon treatment (velocity: p = 0.045, Fig. 3A/B) and was attributed to diminished exploratory behavior.

Next, it was examined whether improvement of memory was associated with decreased neuroinflammation in the brain. The degree of microgliosis and astrocytosis were analyzed in cortex and hippocampus by Iba1/Congo Red and GFAP staining (Fig. 3B; Supplementary Fig. 4, respectively). As expected, significant genotype-dependent differences were observed between Wt and APP/PS1 animals (Iba1: p < 0.0001; GFAP: p < 0.0001; Fig. 3B, Supplementary Fig. 4, respectively) and no differences between treated and untreated Wt mice. A significant reduction of GFAP as well as Iba1 staining was observed in the cerebral cortex in treated APP/PS1 mice (Iba1: p = 0.0094; GFAP: p < 0.0001; Fig. 3B; Supplementary Fig. 4, respectively). In addition, no differences in the ratio of Iba1 stained microglia counts surrounding amyloid plaques to the area of the respective Congo Red stained amyloid plaques was found between treated and untreated APP/PS1 mice in the cerebral cortex (Fig. 3C). In the hippocampal area Iba1 and GFAP levels were significant between Wt and APP/PS1 animals (Iba1: p < 0.0001; GFAP: p < 0.0001; Fig. 3B; Supplementary Fig. 4, respectively). In untreated and treated Wt animals no differences were observed, while untreated and treated APP/PS1 animals showed significant improvement which was less pronounced when compared to the cortical analyses (GFAP: p = 0.0003; Fig. 3B; Supplementary Fig. 4, respectively). However, when investigating the ratio of microglia counts-to-proximal amyloid plaque area in the hippocampus of APP/PS1 animals, a significant reduction in the ratio was examined in treated animals (p = 0.015; Fig. 3C).

Due to the stronger impact of treatment on microglia and astrocyte marker in cortical tissue, the treatment-dependent effect on cortical cytokine and chemokine levels and transcriptome was measured. Although IL-1β and TNFα showed significant differences between APP/PS1 treatment groups, the results had no biological relevance due to the overall low levels of cytokines and chemokines (Supplementary Fig. 2C). Next, the total RNA expression of whole cortical tissue was analyzed by a focused hypothesis-driven analysis (gene enrichment) comparing treated and untreated APP/PS1 mice although the EdgeR analysis did not show differential expressed transcripts after FDR (Fig. 3D/E). Three pathways were significantly altered: endocytosis, lysosome, and glycerophospholipid metabolism (p = 0.032, p = 0.048 and p = 0.051, respectively; Fig. 3D). As endocytosis and lysosomal activity are particularly associated with glial cells, the dataset was reanalyzed for specific brain cell markers. Microglial and astrocytic cell marker GFAP, TMEM119, Aif1, Olfml3 and Ccl3 were shown to be differentially expressed in treated APP/PS1 mice (p_FDR_ = 0.046, p_FDR_ = 0.036, p_FDR_ = 0.005, p_FDR_ = 0.010 and p_FDR_ = 0.002, respectively; Fig. 3E).

### Multi-Omics integration for identification of crucial parameters

**Figure 4:**
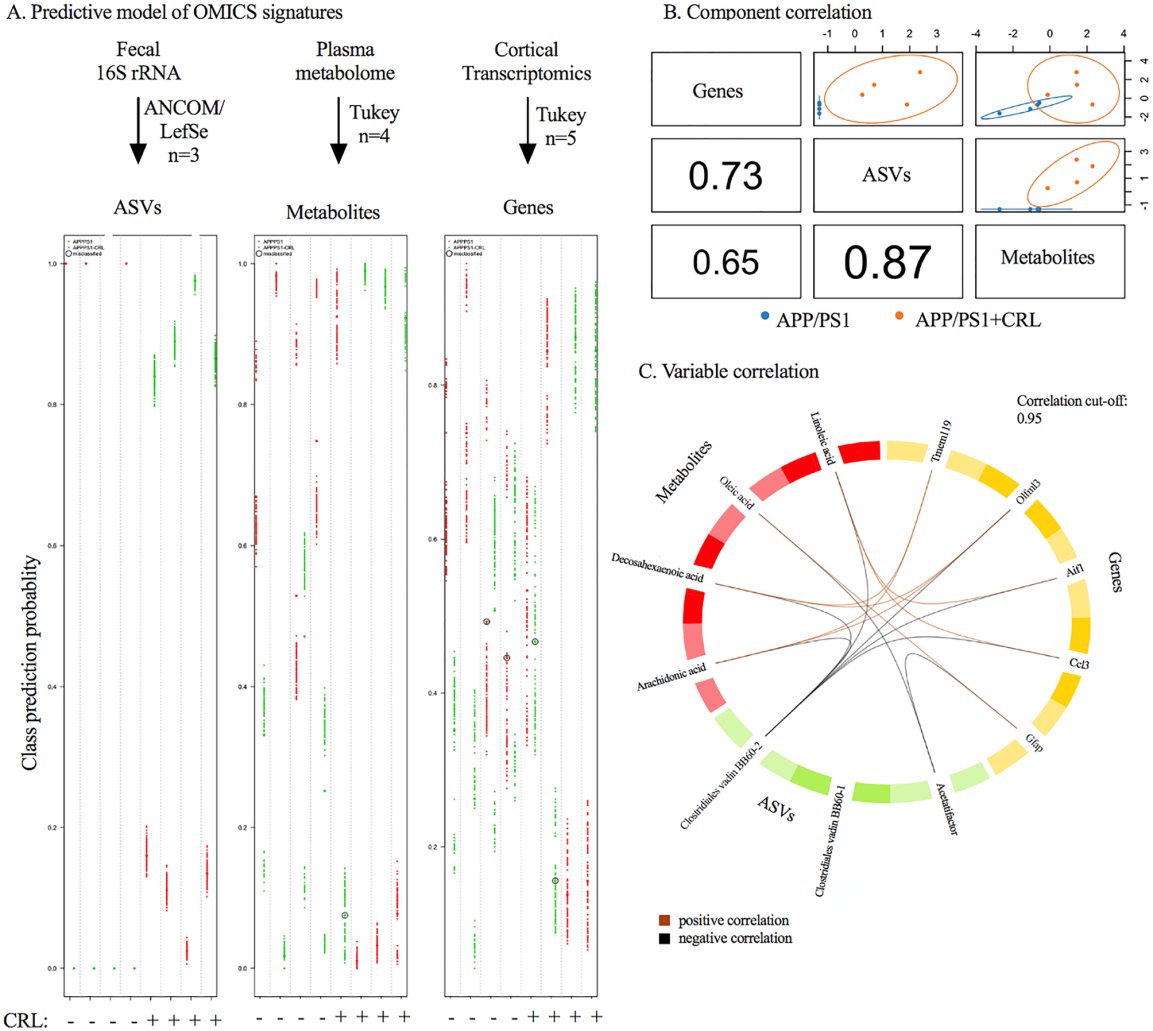
Multi-Omics integration via rdCV-RF and DIABLO mixOmics analysis. **A** rdCV-RF analysis displayed as swim lane plot. Each swim lane displays one sample. Circled points indicate misclassified components. Each component was assessed by 200 double cross-validations to analyze class probabilities. **B** Component correlation of each of the three datasets determined by DIABLO analysis, which maximized the correlated information. **C** Variable correlation displayed in Circos plot with correlation cut-off of 0.95. Two separate pathways were identified. Positive correlation (brown), negative correlation (black).

A total of three Omics datasets including metagenomic (fecal 16S rRNA), metabolomic (plasma untargeted metabolomics) and transcriptomic data (cortical RNAseq) were analyzed to investigate global treatment effects in APP/PS1 mice. The focus of our integrated omics analysis was guided by the significant variables determined in the gut metagenomic, plasma metabolomic, and cortical transcriptomic datasets described above. However, to assess the variables’ dependencies and strength in relation to treatment, repeated double-cross validation with random forest (rdCV-RF) was applied on the identified significant features to prevent statistical overfitting as described by Liu et al. (Fig. 4A)^29,30^. The rdCV-RF analysis revealed that 100% (determined by division of misclassified parameters and total number of parameters) of amplicon sequence variants (ASVs), 75% of metabolites and 20% for transcripts were correctly classified highlighting the weakness of the selected transcriptomic parameters (Fig. 4A). This was further emphasized by the under the curve (AUC) values, which were used to evaluate model performance post rdCV-RF analysis. ASVs and metabolite datasets resulted in an AUC of 0.8 to 1, suggesting good model performance, while the transcriptomics dataset only reached 0.62 suggesting again weak performance in accordance with the EdgeR analysis (Fig. 4A/B).

When determining the correlation between the three datasets, the strongest correlation was observed between ASVs and metabolites, while metabolites and transcripts showed the weakest interaction (Fig. 4B). The correlation between variables was examined by Data Integration Analysis for Biomarker discovery using a latent component method for Omics (DIABLO, correlation cut-off of 0.95), which revealed two separated correlated pathways: (1) *Clostridiales vadin BB60 group uncultured bacterium 2* showed significant negative correlations with linoleic, arachidonic and docosahexaenoic acid and genes associated with microglial gene expression; while (2) *Acetatifactor* was negatively correlated with GFAP and oleic acid (Fig. 4C). Nonetheless, the interpretation of correlations to RNA transcripts must be further validated due to the weakness of the transcriptomic dataset.

In summary, CRL treatment altered the gut metabolome and microbial composition, normalized peripheral UFA levels and increased fatty acid transport, while reducing AD-like pathology and improving aberrant behavior.

### Validation of CRL’s induced gut changes and their contribution to memory improvement

**Figure 5:**
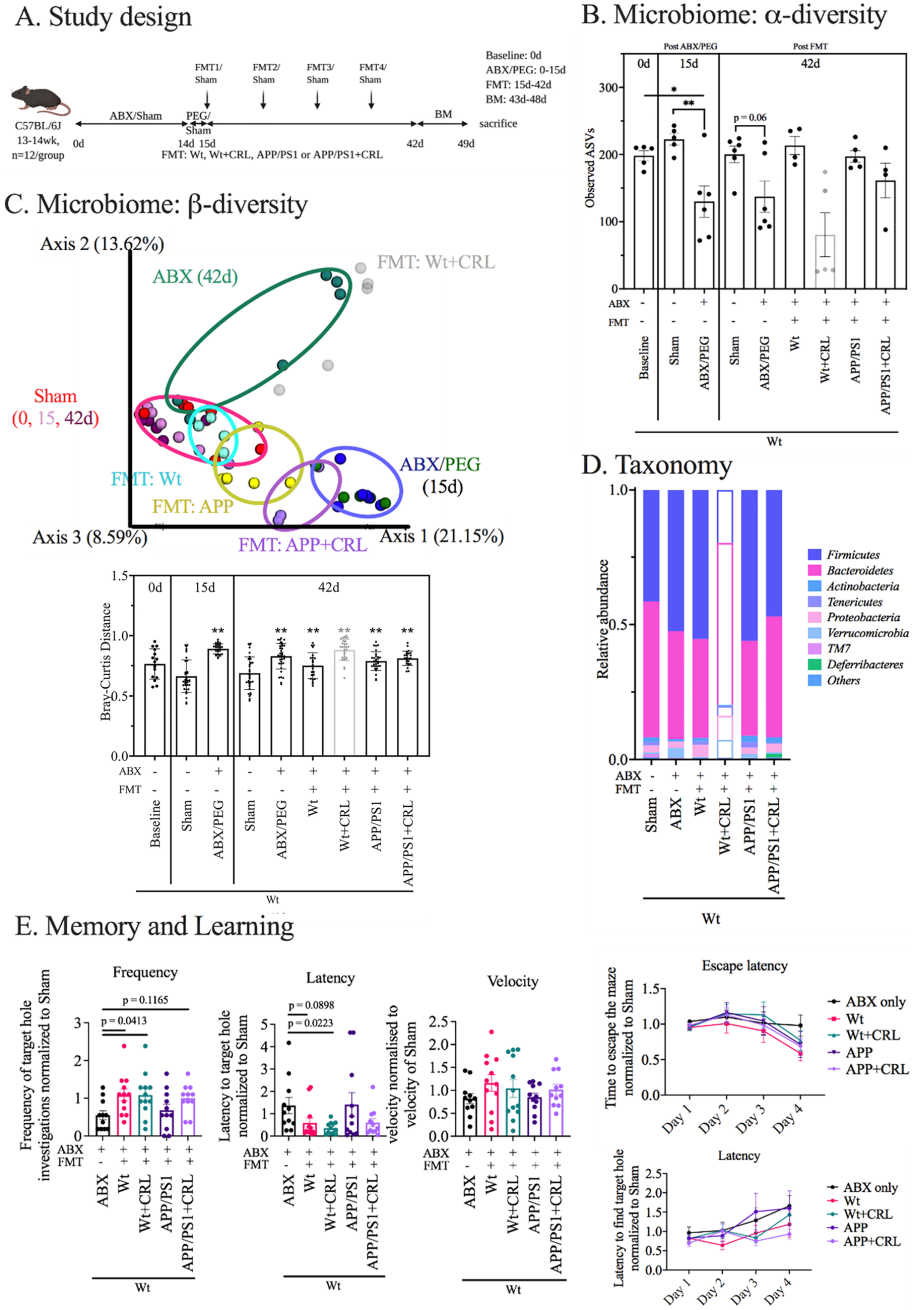
Transferability of results via FMTs in AIMD WT mice. **A** Study design. **B** α-diversity measured as observed ASVs indicated depletion post antibiotics and bowel cleanse and reshaping post FMT. **C** β-diversity measured by Bray–Curtis distance. While Sham groups co-located and ABX and PEG groups co-located, animals receiving FMTs were distinct but located separately. The ABX group recovered partially. For analysis, the group receiving Wt + CRL FMT (abnormal α- and β-diversity) was fainted throughout microbial analysis, although no impact on behavioral data was observed. **D** Taxonomic analysis of phyla levels in fecal samples post FMT. Besides in animals receiving Wt + CRL, no major differences were found between groups. **E** Barnes maze experiment for examination of differences in memory and learning. Results were normalized to sham group to reduce complexity of analysis. Analysis revealed that AIMD WT mice receiving none, or FMT from untreated APP/PS1 mice, performed similar or worse than all other groups. Significance was determined as in prior experiments of microbiome and Barnes maze analysis. The probe trial results were compared to ABX group only. Significance: *p < 0.05, **p < 0.01, ***p < 0.001.

To categorially analyze whether the observed changes of AD-like pathology in APP/PS1 mice from *Study 1* were primarily driven by the gut-induced changes through CRL treatment; fecal samples of all groups of *Study 1* were collected from treatment week 5 to 8. The fecal matter of each group was pooled and stored as live stocks. These live stocks were then transplanted into AIMD Wt mice, which exhibited 50% reduced amounts of observed ASVs in fecal samples prior to the fecal transplants (Fig. 5A/B). After transplantation, the α-diversity index (observed ASVs) at day 42 showed that animals receiving FMTs from Wt animals (untreated) and APP/PS1 animals (treated and untreated) exhibited comparable ASVs when compared to the Sham group (Fig. 5B). In contrast, the ABX group, which received sham FMTs, maintained a reduction of 30 to 40% observed ASV levels after 42 days. This suggested that the gut microbiome was successfully disturbed after treatment (Fig. 5B). Fecal β-diversity of the Sham group at 0, 15 and 42 days co-located, while the ABX and PEG group (day 14 and 15, respectively) showed a significant dimensional separation of the Bray–Curtis distance from the Sham group in accordance with the previous results (Fig. 5C). After 42 days, β-diversity in mice that received antibiotics and sham FMT (ABX) or antibiotics and FMT from treated Wt mice (Wt + CRL) was separated from the Sham group, while antibiotic-depleted mice receiving fecal matter from APP/PS1, APP/PS1 + CRL and Wt mice shifted back towards the Sham group. This suggested partial recovery of these groupsConsequently, this group was excluded and labelled by light grey data points/bars in the microbial analysis (Fig. 5B/C/D). from AIMD. The most prominent taxonomical difference between ABX, WT + CRL and APP/PS1 groups compared to the other three groups was the increased abundance of *Verrucomicrobia* and *Firmicutes*, and decreased *Bacteroidetes* (Fig. 5D). The overall unexpected results of the Wt + CRL group led to additional analyses that showed abnormal phyla levels, enlarged ceca and increased levels of *δ-Proteobacteria* (Fig. 5B–D; Supplementary Fig. 5A–C). Donor and recipient fecal material did not exhibit major differences between phyla except for the excluded Wt + CRL recipient group (Supplementary Fig. 5C). Altogether, this suggested a present infection in this group that may have occurred during FMT administration.

After microbial depletion and recolonization, the respective microbiomes were analyzed in relation to cognitive performance since treatment with antibiotics and the associated dysbiosis have been reported to inhibit learning and long-term memory^31^. All groups were normalized to Sham and compared to the ABX group. None of the groups showed overt differences during acquisition trials in the Barnes maze (Fig. 5E; Supplementary Fig. 5E). During the probe trial AIMD Wt mice receiving FMT’s from untreated APP/PS1 mice showed impaired memory (APP/PS1; frequency: 68.33%, p = 0.919; latency: 140.96%, p = 0.661; Fig. 5E; Supplementary Fig. 5D) like AIMD Wt mice not receiving FMTs (ABX; frequency: 55%; latency: 136.77%; Fig. 5E; Supplementary Fig. 5D). Mice receiving FMTs from CRL treated APP/PS1 mice exhibited a trend for improvement (APP/PS1 + CRL; frequency: 100%, p = 0.116; latency: 60.24%, p = 0.257; Fig. 5E; Supplementary Fig. 5D) like mice receiving fecal matter from untreated animals (Wt; frequency: 108.47%, p = 0.041; latency: 58.83%, p = 0.067). Despite the infection, mice receiving FMTs from Wt + CRL mice performed as well as untreated Wt mice in the Barnes maze (Wt + CRL; frequency: 108.47%, p = 0.041; latency: 35.71%, p = 0.022; Fig. 5E; Supplementary Fig. 5D). Hence, we established that alteration of the microbial and metabolic composition by CRL treatment contributed to mitigation of AD-like pathology and normalized aberrant behavior in treated APP/PS1 mice.

## Discussion

Previous studies have shown that dietary interventions can lead to an altered gut microbial composition and shifts in gut metabolites with potential benefits to the host’s health^32^. However, it is still unclear how these alterations can evoke benefits at sites distant to the gut including brain-related functions. Understanding the underlying mechanisms is an urgent need in microbiome research to determine the potential for microbiome-targeting treatment approaches in central nervous system diseases. To distinguish between cause-and-effect observations in this area of research, which will enhance clinical translation, the complex system’s networks and pathways must be understood in depth. Therefore, *Study 1* examined the evoked treatment-dependent alterations in the gut microbial composition and metabolome, and propagated effects to distal body sites in APP/PS1 compared to Wt mice. In addition, we assessed in *Study 2* the causal relationship between CRL-induced gut environmental changes and improvement in memory via FMT.

In *Study 1*, CRL treatment altered the microbial and metabolic composition in the gut without affecting gut barrier integrity as we previously showed^15^. The selected CRL dose was determined by translation of an approved human Creon dose, which is administered in patients with cystic fibrosis or pancreatic insufficiency^33^. Three microbial genera distinguishing CRL treated from untreated APP/PS1 mice were observed: two *Clostridiales vadin BB60 uncultured* and *Acetatifactor* genera. The cecal metabolomics analysis showed elevated fatty acid release upon treatment. *Acetatifactor* was discovered in an obese mouse and was seen to occur in response to high-fat diets further supporting CRL’s activity in the gut^27,34^. This genus has been suggested to counteract harmful obesity effects by stimulating the GLP-1 secretion, which can improve liver function and enhance glucose and insulin sensitivity as well as host energy metabolism^35,36^. The increased abundance of *Acetatifactor* could have therefore counteracted features that are known to frequently occur in APP/PS1 mice as well as AD patients prior to onset of plaque pathogenesis and cognitive decline^37–39^. The genus *Clostridiales vadin BB60* has been less explored but is associated with homovanillic acid and 5-hydroxyindole acetic acid levels in the brain stem^40^. The increased abundance of all three genera might, therefore, suggest increased communication of the gut-brain axes and together with the elevation of lipid hydrolysis may have contributed to the observed amelioration of AD-like pathology^14,41,42^.

Next, peripheral and brain alterations were investigated to assess whether the treatment-dependent changes were affecting AD-like pathology. While the immunological axis was shown to be treatment-independent, the metabolic axis revealed treatment-dependent changes in certain unsaturated fatty acid levels, which were elevated on average by 65% in untreated APP/PS1 mice but mitigated in treated APP/PS1 mice. However, elevation of ω-3 fatty acids levels in particular had been shown to evoke anti-inflammatory effects and might have been an endogenous mechanism to counteract progressing APP/PS1 pathology^43^. Hence, one might hypothesize that reduction of those levels might harm the host and facilitate progression of the disease post CRL treatment^44^. But since these mice exhibited ameliorated brain pathology, we hypothesized that increased peripheral UFA levels in untreated APP/PS1 mice might have been induced by increased lipolysis and/or reduced liver metabolism rather than an endogenous protective mechanism. It has been shown that increased levels of peripheral FFAs—as observed in untreated APP/PS1 animals—by increased lipolysis reduces BBB integrity, increases insulin resistance, disturbs energy metabolism and can exacerbate neuroinflammation^37,45,46^. Normalization of UFA levels would have therefore contributed to reduction of parameters contributing to AD-like pathology. However, while we reported increased UFA levels in APP/PS1 mice, levels in AD patients have been shown to be decreased^47^. Nevertheless, a study of patients with probable AD reported reduced VLDL cholesterol levels in these patients supporting our finding and emphasizing the potential treatment mechanism of CRL at the selected time-point^48^. In conclusion, a longitudinal study of peripheral and brain fatty acid levels will be necessary to understand whether the elevation in untreated APP/PS1 animals was based on a disease-relevant mechanism at a specific time-point in the disease progression or whether this represented a specific occurrence in the selected mouse model. This will enhance the understanding of whether the treatment-dependent reduction of ω-3 and ω-6 fatty acids contributed to the observed amelioration of brain pathology.

The observed treatment-dependent reduction of microgliosis and astrocytosis supports this hypothesis. Reactive astroglia and microglia populations have been reported to increase synaptic loss in cortical and hippocampal tissue and cognitive decline^49–52^. Interestingly, it has been reported that defective lipid metabolism of astrocytes is reversed by a high-fat diet counteracting neurological deficits in sterol regulatory element-binding protein (SREBP)—cleavage-activating protein (SCAP) depleted mice supporting our selected treatment strategy^53^. Based on the microbial and metabolic results of this study, we propose that CRL treatment might have created a similar environment improving neuroinflammation and cognitive performance as determined in the Barnes maze. Finally, to identify potential drivers of the observed effects and the associated pathways, we conducted a cortical transcriptomic analysis. No significant differences post FDR correction were found. When applying gene enrichment analysis on the significant transcripts prior to FDR correction, endocytosis, lysosomal degradation and glycerophospholipid metabolism were identified as significant pathways. Endocytosis and lysosomal degradation are protective mechanisms of microglial and astrocyte neuroimmune activity, while the glycerophospholipid metabolism was associated with the peripheral results of altered lipid metabolism. The subsequent analysis of cell specific markers of the transcriptomic dataset revealed gene expression differences of microglia and astrocyte transcripts. The reduction in astrocyte marker GFAP suggested a potential reduction in reactive astroglia, while microglia marker TMEM119 (homeostatic marker), Aif1 (activated microglia), Ccl3 (inflammatory marker), and Olfmfl3 indicated a reduction of the reactive microglia population in the cortex^49,54–57^. In future studies, transcriptomic analysis of isolated cell populations such as microglia or astrocytes of both cortex and hippocampus should be applied to prevent transcript dilution by, for instance, neurons, which will enable stronger conclusions. This shortfall was further emphasized in the multi-omics integration analysis, which exposed the transcriptomics dataset’s weak performance, while highlighting the vital role of the determined ASVs and metabolites in the treatment-dependent effects.

In summary, CRL administration in the gut might have reduced AD-like pathology by increased release of a variety of fatty acids, which altered the gut microbiome and metabolome and rebalanced energy metabolism. This might have led to normalization of peripheral fatty acid levels by enhancing liver homeostasis and thereby lipid transport. Consequently, this might have rebalanced the fatty acid composition in the brain and reduced oxidative stress in neurons, which resulted in reprogramming of astrocytes and microglia enhancing neuronal activity and brain performance.

As an alternative hypothesis, we proposed that UFA level reduction represented a consequence of treatment-dependent amelioration of brain pathology. Hence, microbial, and metabolic alterations in the gut might have propagated through a different axis in the brain—such as the neuronal axis. The treatment-dependent increase in cecal short-chain fatty acids might have activated enteric vagal signaling. Enteric vagal afferents project into the nucleus tractus solitarius and dorsal motor nucleus, which can signal via dopaminergic or serotonergic neurons in different areas of the brain such as the hippocampus and cortex^58–60^. As consequence, astrocytes and microglia might have been reprogrammed into the anti-inflammatory subtype by neuronal signaling or respective metabolites travelling to the brain via the vagal route^61,62^. The reduction of neuroinflammation might have in turn recovered lipid transport and normalized peripheral levels of ω-3 and ω-6 fatty acids in APP/PS1 mice.

To finally evaluate whether the beneficial treatment-dependent changes in the gut caused the reduction in AD-like pathology, we investigated whether these beneficial changes were transferable. Wt mice receiving FMT’s from treated APP/PS1 mice exhibited improved memory when compared to ABX mice receiving FMT from untreated APP/PS1 mice or none. This supported the study’s hypothesis that CRL treatment could recover a healthy gut environment, which reduced AD-like pathology in APP/PS1 mice.

These findings demonstrate the potential of digestive enzymes as clinically relevant agents to potentially treat diseases such as AD. CRL treatment in diseases of ageing are particularly interesting since enzyme activity has been shown to decline with increasing age, potentially contributing to dysfunction of glucose and lipid metabolism^63,64^. More experiments need to be conducted to further exploit the pathway leading to the determined beneficial effects and to validate the findings in other AD mouse models and human studies to enhance translatability. Nevertheless, this series of studies may have presented a novel strategy to target and treat neuropathology of AD but also pathologies of other neurodegenerative diseases due to their common association with ageing and inflammation.

## Materials and methods

### Animal models

Animals were kept in the Association for Assessment and Accreditation of Laboratory Animal Care International (AAALAC) accredited vivarium of the Roskamp Institute. Experiments with mice were reviewed and approved by the Institutional Animal Care and Use Committee (IACUC) of the Roskamp Institute before implementation and conducted in compliance with the National Institutes of Health Guidelines for the Care and Use of Laboratory Animals. The design of both animal studies and their connection have been visualized in a study overview figure (Supplementary Fig. 6). Wt and APP/PS1 for *Study 1: CRL treatment study* were obtained from in-house breeding. Wt mice for the *Study 2: FMT study* were also obtained from in-house breeding. Mice were maintained on a 12 h/12 h light/dark cycle and received food and water ad-libitum. All studies have been reported in accordance with the ARRIVE guidelines.

### Study 1: CRL treatment study

Mice (Wt or APP/PS1, 8 months of age, n = 12–15/group) were obtained from an internal breeding protocol, and randomly subdivided into CRL treated and untreated groups. Mice received 5000 *Fédération Internationale Pharmaceutique* (FIP)/kg body weight CRL (300,000 FIP/g, Enzymedica, FL, USA) in drinking water for 2 months, or regular water. The used dose was extrapolated from Creon, a human digestive enzyme treatment for pancreatic insufficiency^33^. CRL has been shown to be stable in water for up to 7 days and hence, water, treated or untreated, was changed twice a week^65^. Beginning four weeks before *Study 1* started nesting and bedding materials were exchanged between all groups and throughout the study within groups twice a week, to normalize endogenous gut microbial variance and to prevent cage effects. Four groups were assessed in *Study 1* (APP/PS1 n = 12, APP/PS1 + CRL n = 12, Wt n = 13, Wt + CRL n = 15). Fecal samples were collected at 0, 4, and 8 weeks of treatment and in addition, three times per week during treatment week 5 and 8 for the subsequent Study 2. In the last two weeks of treatment, mice were trained and assessed for spatial learning and memory in the Barnes maze. On the last day of treatment, gut integrity was assessed. Animals were humanely euthanized after anesthesia with 2% isoflurane (Patterson veterinary, Greeley, CO) in oxygen via cardiac puncture-induced exsanguination and subsequent perfusion with phosphate buffered saline (PBS) in accordance with the approved IACUC protocol. Right hemispheres of the brain were fixed in 4% paraformaldehyde (Sigma, St. Louis, MO) for 24 h for immunohistochemical analysis. The left-brain hemisphere, gastrointestinal tract, and plasma were collected and immediately flash frozen in liquid nitrogen and stored at − 80 °C.

### Study 2: FMT study

For the FMTs, fecal samples were collected from the animals of *Study 1* three times a week between treatment week 5 to 8 for each group. Fecal samples were immediately homogenized 1:5 (w/V) in 0.9% sodium chloride (Sigma, St. Louis, MO) solution containing 10% glycerol (Sigma, St. Louis, MO) to stabilize fecal bacteria during storage and shorten oxygen exposure. Before usage, all samples of the same group were flash thawed in a 37 °C water bath and combined^66^. The fecal pool of each group was strained through a 40 μM nylon filter (Fisher Scientific, Waltham, MA) by centrifugation at 350 g until a viscous layer formed. The cell density of the flow through was measured in a cell counter (APP/PS1: 6.74 × 10^6^/50 μL; APP/PS1 + CRL: 5.04 × 10^6^/50 μL; Wt: 7.47 × 10^6^/50 μL; Wt + CRL: 8.68 × 10^6^/50 μL) and adjusted to 5 × 10^6^ cells/50μL. Samples were divided in 700 μL aliquots, flash frozen in liquid nitrogen and stored at − 80 °C and used for FMT treatment below.

Wt mice (n = 72, aged 13–14 weeks) were randomly subdivided into 6 groups with 12 mice per group (Sham, ABX, FMT: APP/PS1, FMT: APP/PS1 + CRL, FMT: Wt, FMT: Wt + CRL). The bedding and nesting materials were exchanged between all groups four weeks prior to the study as well as within groups twice a week throughout the study to prevent cage effects. Briefly, the strategy used was adapted from Zarrinpar et al., Wrzosek et al. and Kang et al.^67–69^. Mice were treated with an antibiotic cocktail (Neomycin:Ampicillin:Metronidazole:Vancomycin (Sigma, St. Louis, MO) in 1:1:1:0.25 ratio of 200 mg/kg in drinking water containing 2% sucrose^67,69^)—or sucrose only for controls—for two weeks. Water was changed twice a week. In addition, mice received 100 μg Amphotericin B (Sigma, St. Louis, MO) in 5% dimethyl sulfoxide (DMSO, Sigma, St. Louis, MO) or 5% DMSO (Sham) twice a week in water via oral gavage (20 G, 38 mm, 2 mm tip, straight; GavageNeedle.com, Phoenix, AZ) to prevent fungal overgrowth in depleted gut microbial environment^67^. After antibiotic treatment on day 15, mice fasted for 1 h (water and food) followed by 4 oral gavage injections of 200 μL of 400 g/L Macrogol 4000 (Sigma, St. Louis, MO) 20 min apart or water for controls (Sham) to further remove gut microbiota and remove residual antibiotics before FMT^68,69^. Subsequently, all mice fasted for 4 h. Finally, mice received the first FMT with 5.00 × 10^6^ cells in 50 μL per FMT or 10% glycerol in 0.9% NaCl (Sham, ABX) via oral gavage^66,70^. The three subsequent gavages were administered once weekly thereafter. One week after the last FMT, mice were trained and evaluated for Barnes Maze to determine the effect of FMTs on learning and memory. After behavioral testing, animals were humanely euthanized after anesthesia with 2% isoflurane (Patterson veterinary, Greeley, CO) in oxygen via cardiac puncture-induced exsanguination in accordance with the approved IACUC protocol. Subsequently tissues were collected for 16S sequencing analysis.

### Gut integrity

Prior to euthanasia, mice (APP/PS1 n = 6, APP/PS1 + CRL n = 5, Wt n = 3 and Wt + CRL n = 3 from *Study 1*) were starved (food and water) for 5 h. Next, 4 kDa fluorescein isothiocyanate (FITC)-dextran (0.6 mg/g body weight, Sigma, St. Louis, MO) was orally administered via oral gavage. 60 min later mice were humanely euthanized. Following euthanasia, plasma was collected, flash frozen in liquid nitrogen, and then stored at − 80 °C. For analysis plasma samples were diluted 1:5 in PBS pH 7.4. 100 μL of water and 50 μL of sample—or standard (0–40 μg/mL 4 kDa FITC-dextran)—were added to a black 96-well μ-clear bottom plate (Greiner Bio-One, Monroe, NC). Fluorescence was immediately measured at 485/528 nm and FITC dextran concentration was examined in each sample. Data was analyzed with 2-way ANOVA and Tukey multiple comparisons correction and plotted in GraphPad Prism 8 (GraphPad, San Diego, CA).

### Microbiome analysis

DNA (n = 6/group for *Study 1* and for *Study 2*) was extracted from feces (20 − 100 mg/pellet) and cecum (30 mg) samples using the Fast DNA stool mini kit (Qiagen, Germantown, MD) according to the manufacturer’s protocol. Volumes were adjusted to account for lower sample weight. DNA extracts were analyzed by V3-V4 region 16S rRNA gene amplicon sequencing using the Illumina MiSeq sequencing platform (Illumina, San Diego, CA) in collaboration with Prof. Klatt, University of Minnesota, following the Earth Microbiome Project protocols (http://press.igsb.anl.gov/earthmicrobiome/protocols-and-standards/16s/) and our recent publication^15^. In addition, the MicrobiomeAnalyst interface was used to identify differential abundant microbial genera with the LEfSe application^71^. Data was plotted in GraphPad Prism 8 (GraphPad, San Diego, CA), β-diversity plots were acquired in Qiime2 using the Emperor suite^72^.

### Pro-inflammatory cytokine panel

Cortex and plasma cytokine levels (n = 6/group for *Study 1*) were analyzed with the V-PLEX Plus Proinflammatory Panel1 Mouse Kit (MSD, Rockville, MD) according to the manufacturer’s protocol. Plasma and cortex lysate were diluted 1:2 in Diluent 41, added in duplicates and incubated over night at 4 °C. Subsequently cortex cytokines were normalized to protein content of lysates, which were determined with by bicinchoninic protein assay (ThermoFisher, Waltham, MA) analysis. Data was plotted and analyzed via 2-way ANOVA and Tukey correction in GraphPad Prism 8 (GraphPad, San Diego, CA).

### Cholesterol and cholesterol esters of lipoproteins

For assessment of HDL and VLDL/LDL cholesterol and cholesterol ester in plasma the Cholesterol Assay Kit was used (ab65390, Abcam, Cambridge, MA). The assay was performed following the manufacturer’s protocol.

### Cecum metabolomics

Cecal metabolites (n = 4/group for *Study 1*) were investigated by gas chromatography mass spectrometry analysis. 50 mg of cecum were mixed with 50 μL internal standard mix (10 μg/mL 6,6-D2-glucose, 300 μg/mL D6-γ-aminobutyric acid (all from Sigma, St. Louis, MO); 20 μg/mL 2,2,4,4-D4-citric acid, 10 μg/mL 2,3-D2-fumaric acid, 50 μg/mL U-13C-α-ketoglutaric acid, 50 μg/mL 13C3-malonic acid, 500 μg/mL 3,3,3-D3-lactate, 100 μg/mL 13C3-pyruvate, 10 μg/mL D4-succinic acid, 5 μg/mL D5-phenylalanine, 200 μg/mL 2,3-13C-phosphoenol pyruvate, 100 μg/mL 2,4,4-D3-glutamic acid (all from Cambridge Isotope Laboratories, Tewksbury, MA); 2.5 μg/mL D4-β-hydroxybutyric acid (Cayman Chemicals, Ann Arbor, MI), 20 μg/mL D27-myristic acid (Indofine Chemical Company, Somerville, NJ). Cecal content (30 mg) was homogenized in 50 μL PBS. Samples were extracted by protein precipitation through sample adjustment to 80% methanol and incubation at 80 °C for 3 min (200 μL, ThermoScientific, Waltham, MA). Subsequently, samples were incubated at room temperature for 30 min. Next, samples were methoximated with 10 μL of 40 mg/mL methoxamine hydrochloride in pyridine (Sigma, St. Louis, MO) and incubate at 30 °C for 90 min. Finally, samples were trimethylsilylated for 60 min at 40 °C by adding 50 μL of N-Methyl-(N-trimethylsilyltrifluoroacetamide) (MSTFA) and 1% chlorotrimethylsilane (TMCS, Thermo Fisher, Waltham, MA) and subsequent overnight incubation at room temperature. Samples (100 nL: 1 μL split 1:10) were injected and analyzed with a flow of 1.0246 mL/min on the Agilent 7890A (Agilent Technologies, Santa Clara, CA) GC–MS instrument using the Agilent metabolomics protocol. Samples were separated on a Rxi-5 ms fused silica column (30 m × 0.25 mm, 0.25 um, Restek, Bellefonte, PA) in hexane with a temperature gradient separation: 0–5 min 80 °C, 5–8 min 100 °C, 8–33 min 10 °C/min increase to 350 °C. Quality control samples were generated by combining 5 μL of each sample, which were subsequently injected after each batch. Samples were identified with the NIST database in AMDIS (NIST, Gaithersburg, MD) and deconvoluted with XCMS^73^. Data and statistical analysis were performed in MetaboAnalyst, and treatment and control groups combined for determination of treatment-specific effects^74^. Data was plotted in GraphPad Prism 8 (GraphPad, San Diego, CA).

### Plasma metabolomics

Plasma samples (n = 4/group) were sent to Creative Proteomics (Shirley, NY) for sample preparation, untargeted liquid chromatography mass spectrometry analysis in both positive and negative modes, metabolite identification and pathway analysis. Data was plotted and analyzed via 2-way ANOVA and Tukey correction in GraphPad Prism 8 (GraphPad, San Diego, CA).

### Flow cytometry

Flow cytometry analysis of T lymphocytes was conducted according to the protocol of Joshi et al.^75^. In brief, 200 μL of whole blood (n = 6/group for *Study 1*) were diluted 1:10 in red blood cell lysis buffer (RBC, Fisher Scientific, Waltham, MA), centrifuged and pellet resuspended in 500 μL 95% PBS and 5% fetal bovine serum (FBS). 100 μL suspensions were labeled with 0.25 μg of α-CD4-FITC antibody, 0.25 μg of α-CD8a-PE and/or 0.5 μg α-CD3-Cy7 antibody (11–0042-82, 12–0081-82 and 25–0032-80, respectively, Fisher Scientific, Waltham, MA) for 10 min, then 1 mL 95% PBS (Thermo Fisher, Waltham, MA) and 5% FBS (Thermo Fisher, Waltham, MA) were added to dilute samples. The flow cytometry analysis was performed using the Attune® NxT Acoustic Focusing Flow Cytometer (Thermo Fisher Scientific, Waltham, MA, USA) using Attune® NxT software version 2.7 (Thermo Fisher Scientific, Waltham, MA, USA). The population was gated, subdivided by antibody staining and quantified. Data was presented as percentage to absolute count for each channel and plotted and analyzed via 2-way ANOVA and Tukey correction in GraphPad Prism 8 (GraphPad, San Diego, CA).

### Immunohistochemistry

The collected brain hemispheres of cohort 1 (n = 4/group for *Study 1*) were fixed in 4% para-formaldehyde solution for 24 h and subsequently dehydrated and embedded in paraffin. Brain hemispheres were cut into 9 μm Sects. (4 animals, 4 sections per hemisphere; 2 consecutive sections and 2 consecutive Sects. 90 μm apart) and deparaffinized followed by rehydration through Histo-Clear (Fisher Scientific, Waltham, MA) and ethanol gradient then treated with 0.3% hydrogen peroxide to reduce background staining. Sections were washed in PBS and incubated in blocking serum before an overnight incubation at 4 °C in a 1:8000 dilution of GFAP (Polyclonal Rabbit Anti-GFAP, DAKO, Z0334) or 1:2000 dilution of Iba1 antibody (Iba1; Polyclonal Rabbit Anti-Iba1, Abcam, Cambridge, MA). Next, the sections were washed with PBS and placed in secondary antibody Vectastain Elite ABC reagent (Vector Laboratories, Inc., Burlingame, CA) for 30 min. Iba1 slides were then stained with Congo Red for 10 min to stain Aβ plaques. Then, all slides were incubated in 3,3′-diaminobenzidine (DAB) for 1–3 min. Subsequently, tissue sections were counterstained with hematoxylin, dehydrated through an ethanol gradient as well as Histo-Clear and then mounted to coverslips. Once the slides were dry, three random images at 20 × of the hippocampal area spanning CA1, CA2 and CA3 and cortical area spanning M1, S1, LPtA, MPtA, V2MM were taken (without overlap) per section using an Olympus BX63 Intelligent microscope and quantified with ImageJ. In addition, six images per section of 40 × magnification of microglia surrounding Congo Red stained plaques in both hippocampus and cortex within the same area were taken and plaque size area analyzed with ImageJ. In addition, microglia that directly surround the associated plaque area were counted and the ratio between plaque area and count assessed. Data was plotted and analyzed via 2-way ANOVA and Tukey correction in GraphPad Prism 8 (GraphPad, San Diego, CA).

### Barnes maze

To analyze memory and learning the Barnes maze was used with 18 equally spaced holes around the outer perimeter of the maze (n = 11–15 mice/group for *Study 1*; n = 11–12 mice/group for *Study 2*). The target hole had a box positioned directly beneath it that allowed the mice to exit the maze. Distinct visual cues were positioned on each of the four walls. Mice were trained for 4 days (3 min/trial/day) to use the cues to locate the target hole and escape the maze, which was achieved when the mouse entered the target box positioned under the target hole. On day 5, the target box was removed to evaluate for learning and spatial memory in a 90-s probe trial. Each trial was tracked and recorded using EthoVision XT 14 software. Data plotting and statistical analysis was performed in GraphPad Prism 8 (GraphPad, San Diego, CA) with 3-way ANOVA testing for acquisition and one- or two-way ANOVA dependent on the respective parameter on the probe day with post-hoc multiple comparisons.

### Transcriptomics

Cortex (n = 4/group for *Study 1*) was separated from one frozen hemisphere and immediately transferred into 1 mL of TRIzol (Fisher Scientific, Waltham, MA) under RNase free conditions. Samples were disrupted by ultrasonication. One hundred μL of 1-bromo-3-cholorpropane reagent (Sigma, St. Louis, MO) were subsequently added and samples mixed and centrifuged at 12,000 g for 15 min. Finally, RNA was precipitated by addition of 500 μL ice-cold isopropanol and washed with 75% ethanol in DEPC water (Fisher Scientific, Waltham, MA). The air-dried pellet was resuspended in DEPC water and RNA concentration measured at Cytation 3 (BioTek, Winooski, VT). At least 500 ng RNA was subsequently sent to GENEWIZ LLC (South Plainfield, NJ), for sample processing and total RNA sequencing (20–30 million reads). The sequencing data was analyzed using the following applications from the Galaxy platform^76^: FastQC, MultiQC, Trimmomatic, HiSAT2^77^, MarkDuplicates, Feature Counts^78^, Column Join on Collections, annotateMyIDs, edgeR^79^. Gene enrichment analysis was performed with EnrichR^80^. The examined cell-specific markers included: Aldh1l1, Atp1b2, Aqp4, Sox9, Slc4a4, Mlc1, GFAP (astrocytes); Cx3cr1, P2ry12, TMEM119, Aif1, Olfml3, Ccl3, Itgam (microglia); stmn2, Rbfox3, syt1, syn1 (neurons); Pecam1, Tie1 (Endothelia) and Nfasc, Kndc1 (Oligodendroglia).Data was plotted and analyzed via 2-way ANOVA and FDR correction in GraphPad Prism 8 (GraphPad, San Diego, CA).

### Multi-omics analysis

Multi-omics analysis was performed according to the Liu et al. protocol^29^. Briefly, a rdCV-RF analysis method was applied with an inner tuning and outer testing loop of 100 repetitions and subsequent cross-validation with 1000 permutations^30^. This was followed by DIABLO in the R package mixOmics^81^, which was employed for multi-Omics integration to determine variable correlation throughout the three datasets.

### Ethics approval

Experiments with mice were reviewed and approved by the IACUC of the Roskamp Institute before implementation and conducted in compliance with the National Institutes of Health Guidelines for the Care and Use of Laboratory Animals.

## Supplementary Information

Supplementary Figures.

## Data Availability

Authors did not use custom code or software for the described analysis of this manuscript. All data analyzed during this study are included in this published article. The sequencing data (paired end reads in FASTQ) of microbiome sequencing, the respective manifest and metadata files as well as unfiltered taxonomic classification of each study and specimen are available in the Mendeley data repository^82^.

## Abbreviations

AAALAC: Association for Assessment and Accreditation of Laboratory Animal Care International
ABX: Antibiotics
AIMD: Antibiotic-induced microbiome-depleted
AD: Alzheimer’s disease
APP/PS1: APPswe/PS1-dE9
CRL: *Candida rugosa* Lipase
DAB: 3,3′-Diaminobenzidine
ASV: Amplicon sequence variant
DIABLO: Data Integration Analysis for Biomarker discovery using a latent component method for Omics
DMSO: Dimethyl sulfoxide
FBS: Fetal bovine serum
FC: Fold change
FDR: False discovery rate
FIP: *Fédération Internationale Pharmaceutique*
FITC: Fluorescein isothiocyanate
FMT: Fecal matter transplant
GFAP: Glial fibrillary acidic protein
HDL: High-density lipoprotein
IACUC: Institutional Animal Care and Use Committee
Iba1: Ionized calcium-binding adapter molecule 1
LEfSe: Linear discriminant analysis Effect Size
PBS: Phosphate buffered saline
RBC: Red blood cells
rdCV-RF: Repeated double cross validation random forest
SCAP: Cleavage-activating protein
SFA: Saturated fatty acid
SREBP: Sterol regulatory element-binding protein
Wt: C57BL/6J
UFA: Unsaturated fatty acid
VLDL/LDL: Very-low-density lipoprotein/low-density lipoprotein
