# Supplementary material for: Exogenous lipase administration alters gut microbiota composition and ameliorates Alzheimer’s disease-like pathology in APP/PS1 mice
Source: Sci Rep. 2022 Mar 21;12:4797. doi: 10.1038/s41598-022-08840-7 (PMC8938460; doi:10.1038/s41598-022-08840-7)
Supplement: Supplementary file 1 — Supplementary Figures. [file 41598_2022_8840_MOESM1_ESM.pdf]

Exogenous lipase administration alters gut microbiota composition and ameliorates Alzheimer's disease-like pathology in APP/PS1 mice

Ariane Menden[1, 2, *], *Davane Hall*[1], *Coral Hahn-Townsend*[1], *Courtney A. Broedlow*[3], *Utsav Joshi*[1], *Andrew Pearson*[1, 2], *Fiona Crawford*[1, 2, 4], *James E. Evans*[1], *Nichole Klatt*[3], *Stefan Crynen*[1, 2], *Michael Mullan*[1, 2], *Ghania Ait-Ghezala*[1, 2]

[1]Roskamp Institute, 2040 Whitfield Avenue, Sarasota, FL, 34243, United States.
[2]Open University, Walton Hall, Kents Hill, Milton-Keynes, MK7 6AA, UK.
[3]Division of Surgical Outcomes and Precision Medicine Research, Department of Surgery, University of Minnesota, 420 Delaware Street SE, Minneapolis, MN, 55455, United States.
[4]James A. Haley Veterans' Hospital, 13000 Bruce B. Downs Boulevard, Tampa, FL, 33612, United States.

*Corresponding author: amenden@roskampinstitute.org

Email addresses: AM, amenden@roskampinstitute.org; DH, davanehall@gmail.com; CHT, chahn-townsend@roskampinstitute.org; CB, broed015@umn.edu; UJ, ujoshi@roskampinstitute.org; AP, apearson@roskampinstitute.org; FC, fcrawford@roskampinstitute.org; JE, jevans@roskampinstitute.org; NK, klat0037@umn.edu; SC, scrynen@roskampinstitute.org; MM, mmullan@roskampinstitute.org; GAG, gaitghezala@roskampinstitute.org

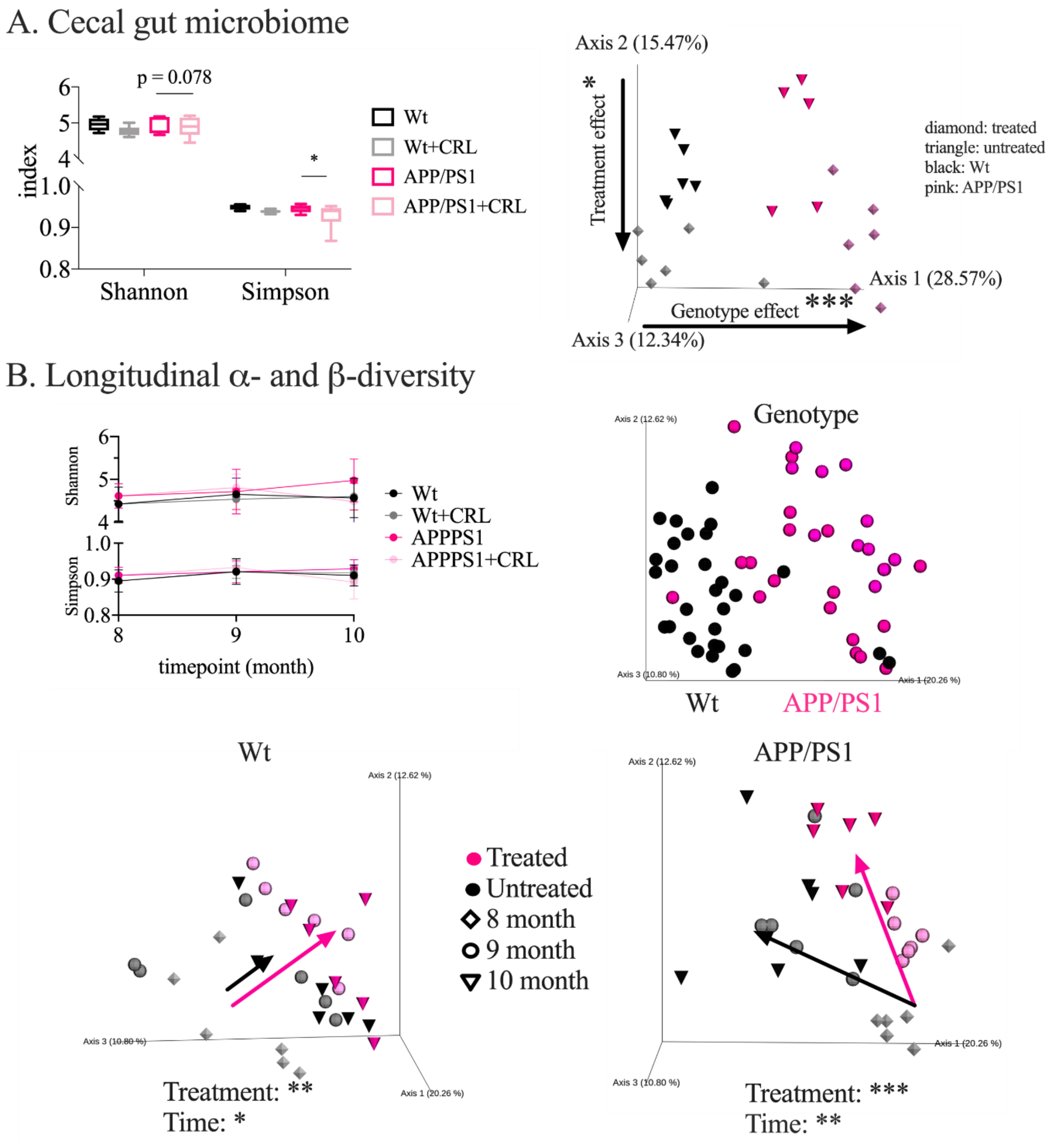

Supplementary figure 1: Cecal and longitudinal fecal microbiota changes. A. Cecal α-diversity was unchanged for the Shannon-index, while the Simpson-index indicated a decrease for APP/PS1 mice post treatment. Cecal β-diversity showed both a significant genotype-dependent shift and a significant treatment-dependent shift, although genotype-dependent shifts were more pronounced. B. Similarly to fecal and cecal α-diversity no major differences were longitudinally determined. However, β-diversity showed a longitudinal genotype-dependent shift but also treatment-dependent effects. While all animals experienced a shift post 1 month independent of treatment status, the treatment-dependent shift appeared more pronounced and continued after 2 months. Significance of α-diversity was determined by Kruskal-Wallis H-test; significance of Bray-Curtis distance was determined by PERMANOVA. Significance: * $p<0.05$, ** $p<0.01$, *** $p<0.001$.

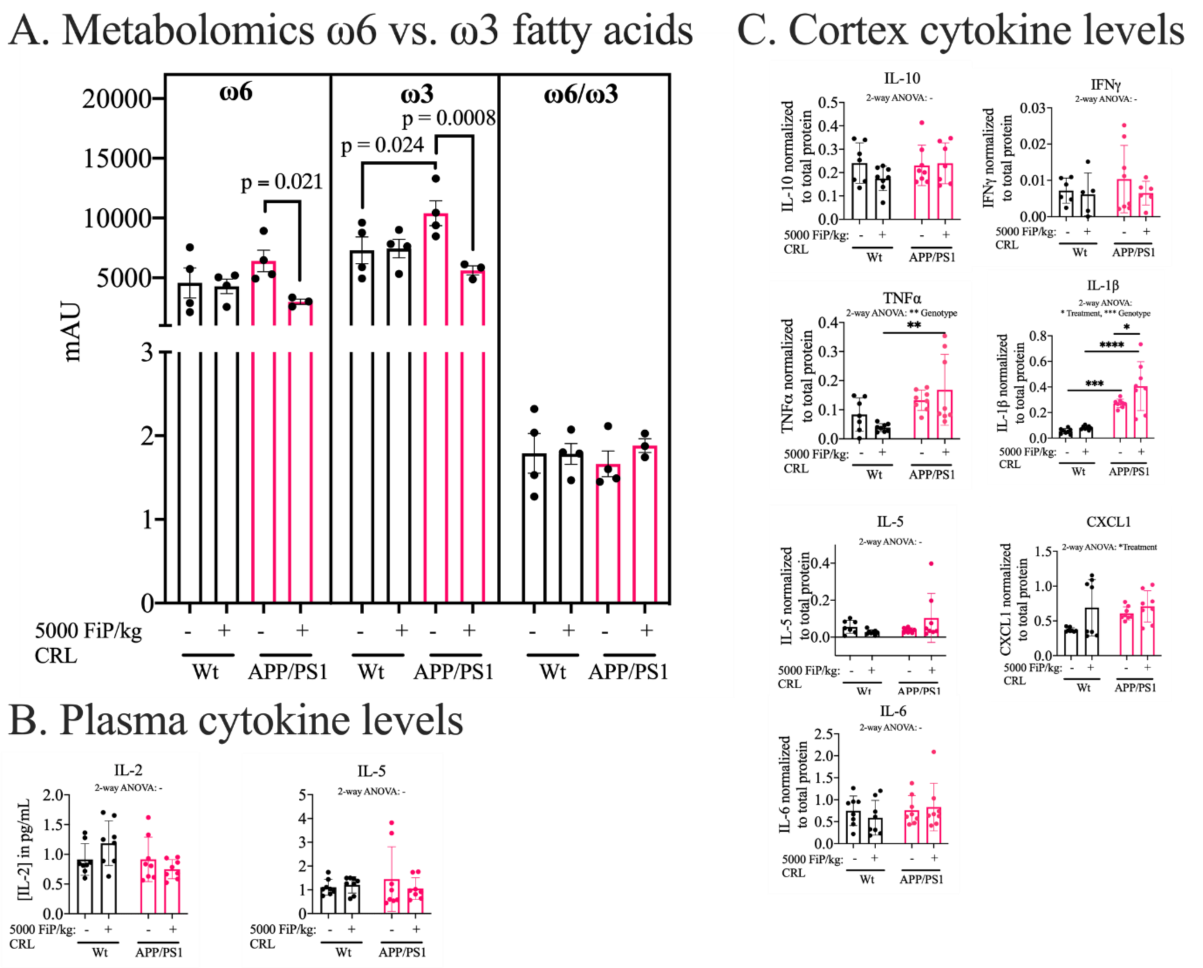

Supplementary figure 2: Peripheral treatment-dependent alterations. A. Metabolomics analysis of ω-3 and ω-6 fatty acid revealed significant increase of ω-3 and ω-6 fatty acids in untreated APP/PS1 mice compared to treated animals and wildtype controls, although ω-3 fatty acid increase was more pronounced. B. Plasma cytokine IL-2 and IL-5 levels were independent of treatment and genotype status. C. Brain cytokine levels did show genotype-specific differences, but no biological relevant treatment-dependent effect. Significance was determined by 2-way ANOVA with Tukey correction. Significance: * $p<0.05$, ** $p<0.01$, *** $p<0.001$, **** $p<0.0001$.

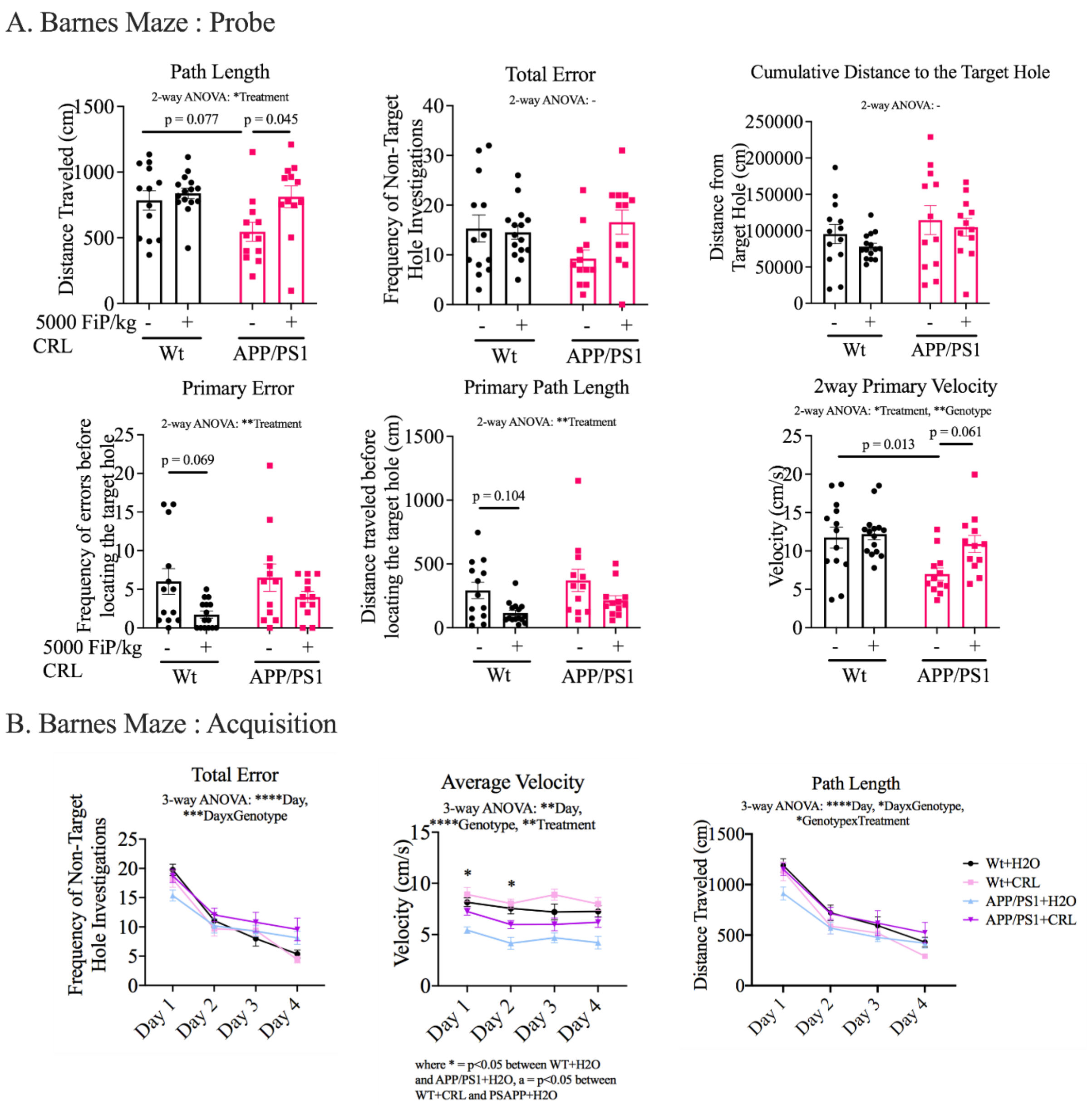

Supplementary figure 3: Barnes maze parameters of wildtype and APP/PS1 treated with CRL. A. Additional probe parameters showing treatment-dependent trends in primary error, primary pathway, and primary velocity (before first target hole interaction). The path length was reduced in untreated APP/PS1 mice similarly to velocity. No significant differences were determined for total error and cumulative distance to target hole. B. The acquisition data shows similarly to the probe significant differences for untreated APP/PS1 mice in average velocity but no difference in path length and total error. Significance of probe data was determined by 2-way ANOVA with Tukey correction, while the acquisition data was analyzed with 3-way ANOVA. Significance: * p<0.05, ** p<0.01, *** p<0.001, **** p<0.0001.

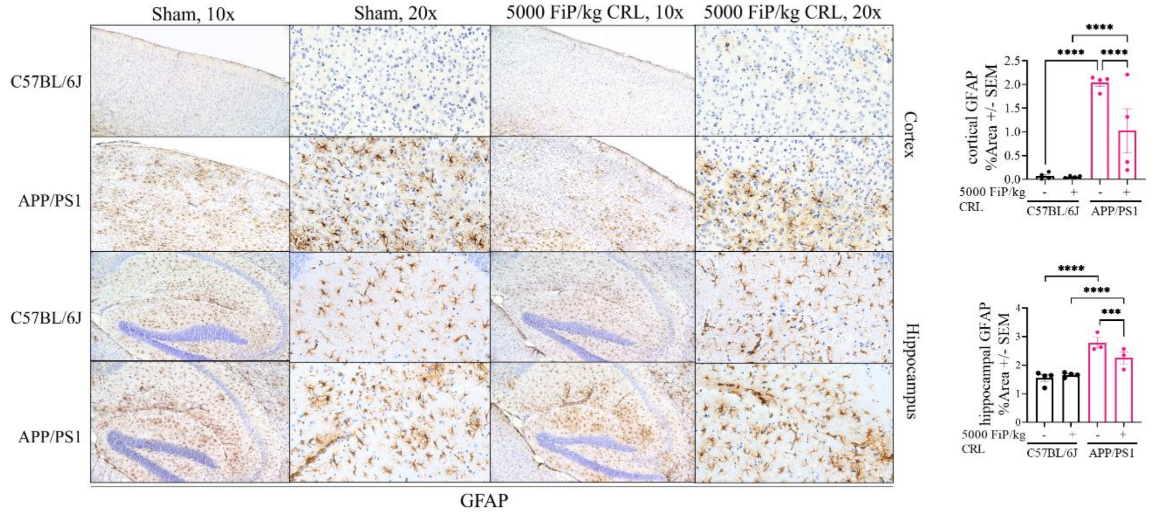

Supplementary figure 4: Immunohistochemical analysis of astrogliosis marker GFAP in cerebral cortex and hippocampal tissue. Investigation of CRL treatment effect on astrocytosis in APP/PS1 and C57BL/6J mice analyzed by quantitation of GFAP staining. Four animals per group with four tissue sections per animal and three images per section were quantified for microgliosis and astrocytosis analysis. Images were analyzed with ImageJ. 2-way ANOVA and Tukey correction were used for immunohistochemical results and 2-way ANOVA and FDR correction for transcriptomics analysis. Significance: *p<0.05, **p<0.01, ***p<0.001.

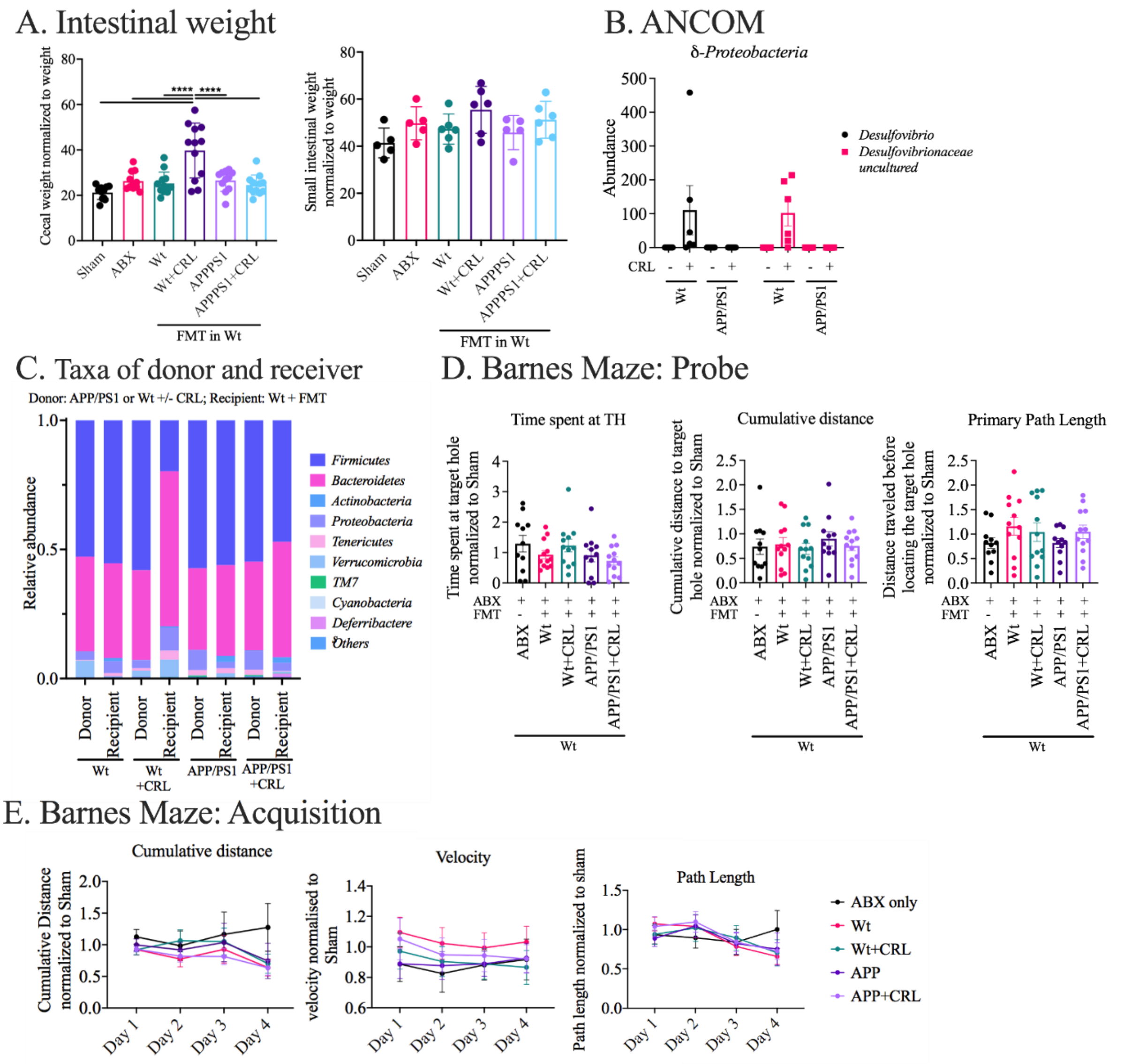

Supplementary figure 5: Fecal matter transplant study infection determination and additional Barnes maze parameters. A. Small intestinal and cecal weight of implanted animals identified enlarged ceca for animals, which received fecal matter from treated wildtype animals. B. ANCOM analysis of taxonomical data revealed a significant increase of δ-Proteobacteria. C. The taxonomy of donor and recipient fecal matter revealed a great imbalance in Wt+CRL recipient mice, which might have been introduced during the oral gavage procedure. D. Additional probe parameters of the Barnes maze analysis revealed no further differences in primary path length, time spent at target hole or cumulative distance. E. The additional acquisition data of the Barnes maze analysis determined no significant difference in cumulative distance, path length or velocity. Significance of cecal and small intestinal weight and probe data were analyzed by 2-way ANOVA with Tukey correction, while acquisition data was analyzed with 3-way ANOVA. Significance: * $p<0.05$, ** $p<0.01$, *** $p<0.001$, **** $p<0.0001$.

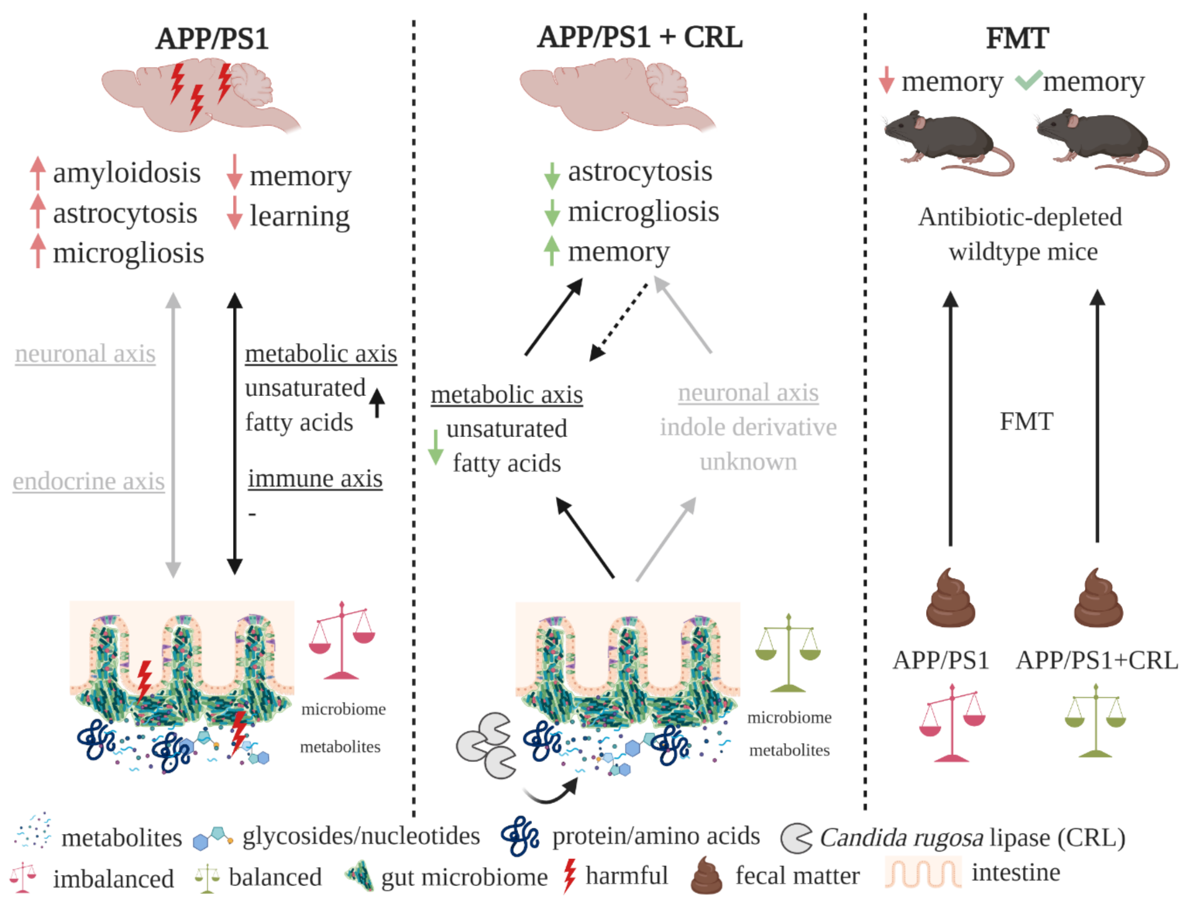

Supplementary figure 6: Study overview. In *Study 1*, 8 months old APP/PS1 mice were treated with 5000 FiP/kg CRL for two months and compared to untreated APP/PS1 littermates and age-matched treated and untreated wild-type littermates. Microbial composition and metabolome, astroglial and microglial activation, plasma fatty acid levels and memory performance improved post treatment. Throughout treatment week 5 to 8, fecal samples of all four groups (Wt, Wt+CRL, APP/PS1 and APP/PS1+CRL) were collected for a subsequent FMT study. The FMT study was conducted in AIMD mice that exhibited memory deficits due to microbiome disturbances. Mice that received fecal matter from APP/PS1 mice that were treated with CRL showed enhanced memory performance, while fecal matter from untreated APP/PS1 mice maintained the memory deficits. This experiment supported the hypothesis that CRL treatment in APP/PS1 mice induced a change in the gut microbiome that enhanced memory.
